# Supplementary material for: Treatment and Survival of Malignant Extracranial Germ Cell Tumours in the Paediatric Population: A Systematic Review and Meta-Analysis
Source: Cancers (Basel). 2021 Jul 16;13(14):3561. doi: 10.3390/cancers13143561 (PMC8305293; doi:10.3390/cancers13143561)
Supplement: Supplementary file 1 [file cancers-13-03561-s001.zip › cancers-1251378-supplementary.pdf]

Appendix 1: Full search strategy in Pubmed and Embase

Pubmed full search

#1 (((tumor[Title/Abstract] OR tumors[Title/Abstract] OR tumour[Title/Abstract] OR tumours[Title/Abstract])))

#2 ((Embryonal [Title/Abstract] OR Chordoma* [Title/Abstract] OR Germinoma* [Title/Abstract] OR Dysgerminoma* [Title/Abstract] OR Seminoma*[Title/Abstract] OR Gonadoblastoma*[Title/Abstract] OR Mesonephroma*[Title/Abstract] OR Endodermal Sinus [Title/Abstract] OR Teratocarcinoma*[Title/Abstract] OR Teratocarcinosarcoma*[Title/Abstract] OR Teratoma*[Title/Abstract] OR Dermoid*[Title/Abstract] OR Struma ovarii[Title/Abstract] OR Trophoblastic neoplasm*[Title/Abstract] OR Choriocarcinoma* [Title/Abstract] OR Gestational trophoblastic disease*[Title/Abstract] OR Germ Cell*[Title/Abstract]))

#3 #1 AND #2

#4 ("Neoplasms, Germ Cell and Embryonal"[Mesh])

#5 #3 OR #4

#6 ((Radia*[Title/Abstract] OR Radiotherap*[Title/Abstract] OR Drug therapy[Title/Abstract] OR Drug therapie*[Title/Abstract] OR adjuvant drug therap*[Title/Abstract] OR chemotherap*[Title/Abstract] OR chemoradi*[Title/Abstract] OR

Operation[Title/Abstract] OR Operative[Title/Abstract] OR procedure*[Title/Abstract] OR Surgery[Title/Abstract] OR Surgical[Title/Abstract] OR Treatment*[Title/Abstract] OR Therapy[Title/Abstract] OR Therapies[Title/Abstract] OR Neoadjuvant Therap*[Title/Abstract] OR Neoadjuvant Treatment*[Title/Abstract]))

#7 (((("Surgical Procedures, Operative"[Mesh]) OR "Radiotherapy"[Mesh]) OR "Chemotherapy, Adjuvant"[Mesh]) OR "Neoadjuvant Therapy"[Mesh])

#8 #6 OR #7

#9 (((Disease-free survival[Title/Abstract] OR DFS[Title/Abstract] OR Progression free survival[Title/Abstract] OR Response Evaluation Criteria in Solid Tumors[Title/Abstract] OR Treatment failure[Title/Abstract] OR Survival[Title/Abstract] OR Outcome*[Title/Abstract] OR Complication*[Title/Abstract])))

#10 "Treatment Outcome"[Mesh]

#11 #9 OR #10

#12 #5 AND #8 AND #11

#13 #12 NOT ("animals"[MeSH Terms] NOT "humans"[MeSH Terms])

FILTER:

- Child: birth-18 years
- Publication dates: from 2000+
- English articles
- Humans

Last performed: 28th of February 2019

Total of 10697 results

Embase full search

#**1** (((tumor:ti,ab,kw OR tumors:ti,ab,kw OR tumour:ti,ab,kw OR tumours:ti,ab,kw)))

#2 ((Embryonal:ti,ab,kw OR Chordoma*:ti,ab,kw OR Germinoma*:ti,ab,kw OR Dysgerminoma*:ti,ab,kw OR Seminoma*:ti,ab,kw OR Gonadoblastoma*:ti,ab,kw OR Mesonephroma*:ti,ab,kw OR 'Endodermal Sinus':ti,ab,kw OR Teratocarcinoma*:ti,ab,kw OR Teratocarcinosarcoma*:ti,ab,kw OR Teratoma*:ti,ab,kw OR Dermoid*:ti,ab,kw OR 'Struma ovarii':ti,ab,kw OR 'Trophoblastic neoplasm*':ti,ab,kw OR Choriocarcinoma*:ti,ab,kw OR 'Gestational trophoblastic disease*':ti,ab,kw OR 'Germ Cell*':ti,ab,kw))

#3 #1 AND #2

#4 'germ cell cancer'/exp

#5 #3 OR #4

#6 ((Radia*:ti,ab,kw OR Radiotherap*:ti,ab,kw OR ‘Drug therapy’:ti,ab,kw OR ‘Drug therapie*’:ti,ab,kw OR ‘adjuvant drug therap*’:ti,ab,kw OR chemotherap*:ti,ab,kw OR chemoradi*:ti,ab,kw OR Operation:ti,ab,kw OR Operative:ti,ab,kw OR procedure*:ti,ab,kw OR Surgery:ti,ab,kw OR Surgical:ti,ab,kw OR Treatment*:ti,ab,kw OR Therapy:ti,ab,kw OR Therapies:ti,ab,kw OR ‘Neoadjuvant Therap*’:ti,ab,kw OR ‘Neoadjuvant Treatment*’:ti,ab,kw))

#7 'surgery'/exp OR 'radiotherapy'/exp OR 'chemotherapy'/exp OR 'neoadjuvant therapy'/exp

#8 #6 OR #7

#9 (((‘Disease-free survival’:ti,ab,kw OR DFS:ti,ab,kw OR ‘Progression free survival’:ti,ab,kw OR ‘Response Evaluation Criteria in Solid Tumors’:ti,ab,kw OR ‘Treatment failure’:ti,ab,kw OR Survival:ti,ab,kw OR Outcome*:ti,ab,kw OR Complication*:ti,ab,kw)))

#10 'treatment outcome'/exp

#11 #9 OR #10

#12 #5 AND #8 AND #11

FILTER:

- Child: birth-18 years
- Publication dates: from 2000+
- English articles
- Humans
- Publication type: Article

Source: Embase and overlapping with medline

Last performed: 28th of February 2019

Total of 1859 results

Appendix 2: Quality assessment of the included articles based on the STROBE Statement (articles 1-16)

STROBE-item	Akyüz 2000	Baranzelli 2000	Schneider 2000	Mann 2000	Terenziani 2001	Stern 2002	Suita 2002	Terenziani 2002	Billmire 2003	Curto 2003	Schlatter 2003	Schneider 2003	Billmire 2004	Güler 2004	Rogers 2004	Marina 2006
1a	●	●	◐	◐	●	●	●	●	●	●	●	◐	●	◐	●	●
1b	●	●	●	●	●	●	●	●	●	●	●	●	●	●	●	●
2	●	●	●	●	●	●	●	●	◐	●	●	●	◐	●	●	●
3	◐	◐	◐	◐	◐	◐	◐	◐	◐	◐	◐	◐	◐	◐	◐	◐
4	●	●	●	●	●	●	●	●	●	●	●	●	●	●	●	●
5	●	◐	●	◐	◐	●	●	●	●	●	●	●	◐	◐	●	●
6a	◐	●	●	◐	◐	●	●	◐	●	●	●	●	◐	◐	●	●

**6b**	◐	◐	◐	◐	◐	◐	◐	◐	◐	◐	◐	◐	◐	◐	◐	◐
**7**	●	●	●	●	●	●	●	●	●	●	●	●	◐	◐	●	●
**8**	◐	◐	●	●	●	●	●	●	●	●	●	●	◐	◐	●	●
**9**	○	○	○	○	○	○	○	○	○	○	○	◐	○	○	○	○
**10**	◐	●	●	◐	●	●	◐	◐	●	●	◐	◐	◐	◐	●	●
**11**	◐	○	◐	●	○	○	○	○	○	●	○	○	○	○	●	●
**12a**	●	●	●	●	◐	◐	●	●	●	●	●	●	●	●	●	●
**12b**	●	●	●	●	◐	◐	●	◐	◐	●	◐	●	○	○	○	●
**12c**	○	○	○	○	○	○	○	○	○	●	○	○	○	○	○	○
**12d**	○	○	○	○	○	○	○	○	○	◐	○	●	○	○	○	○
**12e**	○	○	○	○	○	○	○	○	○	○	○	○	○	○	○	●
**13a**	◐	◐	◐	◐	◐	◐	◐	◐	●	◐	◐	◐	●	◐	◐	◐
**13b**	○	○	●	●	○	●	◐	◐	○	◐	◐	○	◐	○	◐	◐
**13c**	○	○	○	○	○	○	○	○	○	○	○	○	○	○	○	○
**14a**	●	●	●	●	●	●	●	●	●	●	●	●	●	●	●	●
**14b**	●	◐	●	●	●	●	◐	●	●	●	●	◐	●	○	●	○
**14c**	●	●	●	●	●	●	◐	●	◐	●	●	●	◐	◐	●	●
**15**	●	●	●	●	●	●	●	●	◐	●	●	●	●	◐	●	●
**16a**	●	●	●	●	○	○	●	○	◐	●	●	●	○	●	○	●
**16b**	●	●	◐	●	○	○	◐	●	○	●	○	●	○	○	○	●
**16c**	○	○	○	○	○	○	○	○	○	○	○	○	○	○	○	●
**17**	◐	○	◐	◐	○	○	◐	○	○	●	○	●	○	◐	○	●
**18**	●	●	●	●	●	●	●	●	●	●	●	●	●	●	●	●
**19**	○	○	●	○	◐	○	○	◐	◐	◐	○	●	○	○	○	◐
**20**	●	●	●	●	●	●	●	●	●	●	●	●	●	●	●	●
**21**	●	●	●	●	●	●	◐	●	●	●	●	●	●	●	●	●
**22**	○	○	○	○	○	○	○	○	○	○	○	○	○	○	○	○

● = present; ○ = absent; ◐ = unclear/incomplete

<u>Table 2: Quality assessment of the included articles based on the STROBE Statement (articles 17-32)</u>

STROBE-item	Lopes 2008	Lopes 2009	Andrés 2010	Billmire 2014	Cost 2014	Fresneau 2015	Rescorla 2015	Amini 2016	Lopes 2016	Grabski 2017	Shaikh 2017	Terenziani 2017	Duhil de Bénazé 2018	Faure Conter 2018	Frazier 2018	Terenziani 2018
**1a**	●	●	●	●	◐	◐	●	●	◐	●	●	◐	◐	◐	●	●
**1b**	●	●	●	●	●	●	●	●	●	●	●	●	●	●	●	●
**2**	●	●	◐	●	●	●	●	●	●	●	●	●	●	◐	●	◐
**3**	◐	◐	◐	◐	◐	◐	◐	◐	◐	◐	◐	◐	◐	◐	◐	○
**4**	●	●	●	●	●	●	●	●	●	●	●	●	●	●	●	●
**5**	◐	●	●	◐	●	●	◐	●	●	●	◐	◐	●	◐	●	◐
**6a**	●	●	●	●	●	●	●	●	●	●	●	●	◐	◐	●	●
**6b**	●	●	◐	●	◐	◐	◐	◐	◐	◐	◐	◐	◐	◐	◐	◐

**7**	●	●	●	●	●	●	●	●	●	●	◐	●	◐	●	●	●
**8**	●	●	●	●	●	●	●	●	●	●	●	●	◐	●	●	●
**9**	○	○	○	◐	○	○	◐	○	◐	○	○	○	○	○	○	○
**10**	●	●	●	●	◐	●	●	●	●	◐	●	◐	◐	◐	●	◐
**11**	●	○	○	●	●	○	●	●	○	○	○	○	○	●	●	●
**12a**	●	●	●	●	●	●	●	●	●	●	●	●	●	●	●	●
**12b**	●	●	●	●	●	●	●	●	●	●	●	◐	◐	●	●	●
**12c**	○	○	○	○	○	○	○	○	○	○	○	○	○	○	○	○
**12d**	●	●	○	○	○	○	○	○	○	○	○	○	○	○	○	○
**12e**	○	○	○	●	◐	●	●	●	●	●	●	◐	○	●	●	●
**13a**	○	◐	◐	●	○	◐	◐	○	○	◐	●	○	○	◐	◐	●
**13b**	○	○	○	●	○	◐	◐	○	○	◐	●	○	○	●	○	●
**13c**	○	○	○	●	○	○	○	○	○	○	●	○	○	○	○	○
**14a**	●	●	●	●	●	●	●	●	●	●	●	●	◐	●	●	●
**14b**	○	○	◐	●	○	●	◐	○	○	●	◐	●	●	◐	●	●
**14c**	●	●	●	●	●	●	●	●	●	●	●	●	●	●	◐	●
**15**	●	●	●	●	●	●	●	●	●	●	●	●	●	●	●	●
**16a**	●	●	◐	●	●	●	●	●	●	●	●	●	●	●	●	●
**16b**	○	●	○	●	○	○	○	○	●	◐	●	●	○	●	●	●
**16c**	○	○	○	○	●	○	○	○	○	○	○	○	○	◐	◐	○
**17**	○	○	○	◐	◐	●	●	●	●	●	●	◐	○	●	●	●
**18**	●	●	●	●	●	●	●	●	●	●	●	●	●	●	●	●
**19**	●	◐	○	◐	●	○	○	●	○	●	●	◐	●	●	●	●
**20**	●	●	●	●	●	●	●	●	●	●	●	●	●	●	●	●
**21**	●	●	●	●	●	●	●	●	●	●	●	●	●	●	●	●
**22**	○	○	○	○	○	○	○	●	○	○	○	○	●	○	○	●

● = present; ○ = absent; ◐ = unclear/incomplete

STROBE Statement – Checklist of items that should be included in report of observational studies

Title and abstract;

1. Title and abstract

    a. Indicate the study's design with a commonly used term in the title or the abstract

    b. Provide in the abstract an informative and balanced summary of what was done and what was found

Introduction

2. Background/rationale: explain the scientific background and rationale for the investigation being reported

3. Objectives: state specific objectives, including any prespecified hypothesis

Methods

4. Study design: present key element of study design early in the paper
5. Setting: describe the setting, locations, and relevant dates, including periods of recruitment, exposure, follow-up, and data-collection
6. Participants:
    a. Give the eligibility criteria and the sources and methods of selection of participants. Describe methods of follow-up
    b. For matched studies, give matching criteria and number of exposed and unexposed
7. Variables: clearly define all outcomes, exposures, predictors, potential confounders, and effect modifiers. Give diagnostic criteria, if applicable
8. Data sources/measurement: for each variable of interest, give sources of data and details of methods of assessment (measurement). Describe comparability of assessment methods if there is more than one group
9. Bias: describe any efforts to address potential sources of bias
10. Study size: explain how the study size was arrived at
11. Quantitative variables: explain how quantitative variables were handled in the analyses. If applicable, describe which grouping were chosen and why
12. Statistical methods;
    a. Describe all statistical methods, including those used to control for confounding
    b. Describe any methods used to examine subgroups and interactions
    c. Explain how missing data were addressed
    d. If applicable, explain how loss to follow-up was addressed
    e. Describe any sensitivity analyses

Results

13. Participants;
    a. Report numbers of individuals at each stage of study – eg numbers potentially eligible, examined for eligibility, confirmed eligible, included in the study, completing follow-up, and analysed
    b. Give reasons for non-participating at each stage
    c. Consider use of a flow diagram
14. Descriptive data;
    a. Give characteristics of study participants (eg demographic, clinical, social) and information on exposures and potential confounders
    b. Indicate number of participants with missing data for each variable of interest
    c. Summarise follow-up time (eg, average and total amount)
15. Outcome data: report numbers of outcome events or summary measures over time
16. Main results;
    a. Give unadjusted estimates and, if applicable, confounder-adjusted estimates and their precision (eg, 95% confidence interval). Make clear which confounders were adjusted for and why they were included
    b. Report category boundaries when continuous variable were categorized
    c. If relevant, consider translating estimates of relative risk into absolute risk for a meaningful time period
17. Other analyses: report other analyses done – eg analyses of subgroups and interactions, and sensitivity analyses

Discussion

18. Key results: summarise key results with reference to study objectives

19. Limitations: discuss limitations of the study, taking into account sources of potential bias or imprecision. Discuss both direction and magnitude of any potential bias
20. Interpretation: give a cautious overall interpretation of results considering objectives, limitations, multiplicity of analyses, results from similar studies, and other relevant evidence
21. Generalizability: discuss the generalizability (external validity) of the study results

Other information

22. Funding: give the source of funding and the role of the funders for the present study and, if applicable, for the original study on which the present article is based
